# Reducing Post‐Fall Emergency Department Transfer From Residential Aged Care Homes: The Falls Outreach and Residential Mobile Assessment Team (FORMAT) Before‐and‐After Study

**DOI:** 10.1111/1742-6723.70276

**Published:** 2026-05-18

**Authors:** Patrick J. Owen, Andrea Bee, Donna Pattison, Joseph Miller, Liam Hackett, Paul Buntine

**Affiliations:** ^1^ Eastern Health Emergency Medicine Program Melbourne Victoria Australia; ^2^ Eastern Health Clinical School Monash University Melbourne Victoria Australia; ^3^ Eastern Health Residential Inreach Service Melbourne Victoria Australia

**Keywords:** emergency medicine, emergency service, geriatrics, homes for the aged, hospital, hospitalisation, nursing

## Abstract

**Objective:**

We examined whether the 116 emergency department transfers from residential aged care homes prevented by an intervention that provided on‐site assessment and management following a fall led to a detectable shift in system‐level emergency department utilisation.

**Methods:**

This 17‐month before‐and‐after study was conducted at three metropolitan hospital emergency departments of a single health service in Melbourne, Australia. Monthly post‐fall emergency department transfers from 108 residential aged care homes were compared before (1 May 2021 to 30 April 2022) and after (1 May 2022 to 30 September 2022) the intervention using two routinely collected administrative datasets. The primary analysis included patients eligible for the intervention. Sensitivity analyses examined patients ineligible for the intervention and a season‐matched before period (1 May 2021 to 30 September 2021).

**Results:**

Mean (SD) monthly post‐fall emergency department transfers from residential aged care homes among patients eligible for the intervention were 92.2 (14.9) before and 74.2 (7.2) after implementation (mean difference [95% CI]: 18.0 [2.9, 33.1], *p* = 0.023; season‐matched: 28.2 [10.3, 46.1], *p* = 0.007). Monthly post‐fall emergency department transfers from residential aged care homes among patients ineligible for the intervention did not significantly differ before and after the intervention (mean difference [95% CI]: 1.8 [−3.0, 6.7], *p* = 0.434; season‐matched: 1.8 [−5.1, 8.7], *p* = 0.566). We detected 78% (*n* ≈ 90) of the verified 116 emergency department transfers prevented.

**Conclusion:**

On‐site assessment and management following a fall at residential aged care homes may reduce system‐level emergency department utilisation. Examining sustainability and cost‐effectiveness appears warranted.

## 1 Introduction

Worldwide, people are living longer, and the number of people aged over 60 years in 2020 (1 billion) is expected to more than double by 2050 (2.1 billion) [1]. Population ageing has subsequently led to novel challenges for healthcare systems worldwide. For example, over 185,000 Australians now live in permanent residential aged care homes [2]. Residents in these settings often have more complex medical needs than their community‐dwelling counterparts, which may in part explain comparatively higher rates of hospitalisation [3]. Falls are common among residents from residential aged care homes and often result in emergency department transfers [4, 5]; however, the appropriateness of these transfers is debated [4]. Therefore, it is critical that strategies be identified to determine the appropriateness of emergency department transfer following a fall among this susceptible population group.

Leveraging existing healthcare services embedded within residential aged care homes, such as the Victorian Residential In‐reach Service [6] or New South Wales Aged Care Rapid Response Team [7], may represent a viable strategy to assess and manage falls on site. We recently demonstrated the implementation utility of a specialised fall assessment team to complement an existing geriatric‐led assessment service embedded within 108 Victorian residential aged care homes [8]. Specifically, we showed that 94% (*n* = 116/123) of residents who experienced a fall could be assessed and managed on site, and our qualitative content analysis among 40 consumers revealed themes of general satisfaction, compliments for staff, and acknowledgement of comprehensiveness [8]. Given the promising results from our initial evaluation [8], the intervention was continued without modification. However, whether our intervention led to changes in system‐level emergency department utilisation remains unknown.

The aim of the current study was to determine whether the 116 emergency department transfers from residential aged care homes prevented by an intervention that provided on‐site assessment and management following a fall led to a detectable shift in system‐level emergency department utilisation. Specifically, we developed a simple search strategy using routinely collected data that enabled the comparison of post‐fall emergency department transfers among patients from residential aged care homes before and after the intervention. The primary analysis included patients eligible for the intervention. Sensitivity analyses examined patients ineligible for the intervention and a season‐matched before period.

## 2 Methods

### 2.1 Study Design and Setting

This 17‐month before‐and‐after study was registered with the Eastern Health Human Research Ethics Committee (ID: QA22‐035‐86089) and reported following the STROBE statement (Supplement A) [9]. Per study conducted in line with the National Statement on Ethical Conduct in Human Research (Sections 2.3.9 and 2.3.10) [10], a waiver of consent was approved for the collection and use of retrospective de‐identified patient data. Data were collected from 1 May 2021 to 30 September 2022 across three metropolitan hospital emergency departments (Box Hill Hospital: 65,050 presentations per year; Maroondah Hospital: 49,182 presentations per year; and Angliss Hospital: 39,953 presentations per year) [11] of a single health service in Melbourne, Australia. Per the Australian Bureau of Statistics *Index of Relative Socio‐economic Advantage and Disadvantage* [12], these hospitals all represent socio‐economically advantaged geographical locations (seventh decile or greater).

### 2.2 Participants

Patients were those who had a post‐fall emergency department transfer from one of the 108 government‐funded residential aged care homes within the catchment area of the three included emergency departments.

### 2.3 Variables

The primary outcome was monthly post‐fall emergency department transfers from residential aged care homes before (1 May 2021 to 30 April 2022) and after (1 May 2022 to 30 September 2022) the intervention.

The intervention was exposure to the *Falls Outreach and Residential Mobile Assessment Team* (FORMAT) initiative, developed to expand the scope of the existing Eastern Health Residential Inreach Service [13] to enable the provision of medical and nursing assessment and on‐site management to residents from residential aged care homes who experienced a fall. The development and implementation of the intervention are detailed elsewhere [8]. In brief, the team received 2 h of emergency medicine educational training on head injuries and wound management (Supplement B). The team included a senior nurse (clinical nurse consultant), as well as a senior (geriatrician) or junior (geriatric registrar) doctor during daytime hours. All residents from residential aged care homes who experienced a fall in the past 24 h were eligible for initial telephone screening by the team. Patients could be referred to the team by residential aged care facility staff, general practitioners, ambulance services, family members, or the Victorian Virtual Emergency Department [14]. Exclusion criteria were: (1) suspected long bone fracture, (2) new neck pain, (3) wound closure requiring sedation, (4) suspected first seizure, (5) active or ongoing resuscitation requirements, or (6) declined involvement. These criteria were developed via consensus between an emergency physician (P.B.), geriatrician (A.B.), and clinical nurse consultant (D.P.), given that the care of these patients would likely benefit from emergency department transfer. Notably, residents with pre‐existing antiplatelet or anticoagulant prescription medication were not excluded. Among eligible patients, the team conducted an on‐site assessment at the residential aged care home. These assessments typically occurred shortly after the initial telephone screening, within the same clinical shift. On‐site care included a structured medical and nursing assessment, evaluation of injuries sustained during the fall, and provision of immediate management where appropriate (e.g., wound care not requiring sedation, monitoring of head injury symptoms, pain management and development of a short‐term care plan).

### 2.4 Data Sources and Measurement

Patients who had emergency department transfers from residential aged care homes were identified by cross‐checking the Victorian Emergency Minimum Dataset [15] and a separate Eastern Health Residential Inreach Service [13] dataset during a 1‐month period (January 2022). A chart audit was then performed to determine an optimal search strategy using only data coded within the Victorian Emergency Minimum Dataset [15]. The final search strategy included the following terms: ‘presenting condition’ field was: ‘fall’ OR ‘collapse – conscious’ OR ‘collapse – unconscious’ OR ‘dislocation’ OR ‘fracture’ OR ‘head injury’ OR ‘soft tissue injury’. Among these patients, those who were eligible for the intervention were determined by excluding the term ‘fracture’ from the ‘diagnosis desc’ field.

### 2.5 Bias

Risk of sampling bias was explored by conducting sensitivity analyses that examined differences in post‐fall emergency department transfers from residential aged care homes before and after the intervention using: (1) patients ineligible for the intervention, and (2) a season‐matched before period (1 May 2021 to 30 September 2021).

### 2.6 Study Size

No a priori sample size calculation was performed. Rather, the study size was that of convenience whereby all patients during the 12‐month period before (selected to establish a stable baseline trend of post‐fall emergency department transfers from residential aged care homes with minimal seasonal variation) and 5‐month period after the intervention commenced (selected to match the timeframe in which the intervention prevented 116 post‐fall emergency department transfers from residential aged care homes) [8] were included.

### 2.7 Quantitative Variables

Frequency data were collapsed from daily to monthly to account for fluctuations in emergency department transfers from residential aged care homes.

### 2.8 Statistical Methods

All analyses were conducted in Stata (v19, StataCorp, College Station, TX, USA). An independent *t*‐test compared monthly post‐fall emergency department transfers from residential aged care homes before and after the intervention. The primary analysis included patients eligible for the intervention. Sensitivity analyses examined patients ineligible for the intervention and a season‐matched before period (1 May 2021 to 30 September 2021). No analyses had missing data. An *α* of 0.05 was adopted for all analyses.

## 3 Results

### 3.1 Participants and Descriptive Data

**FIGURE 1:**
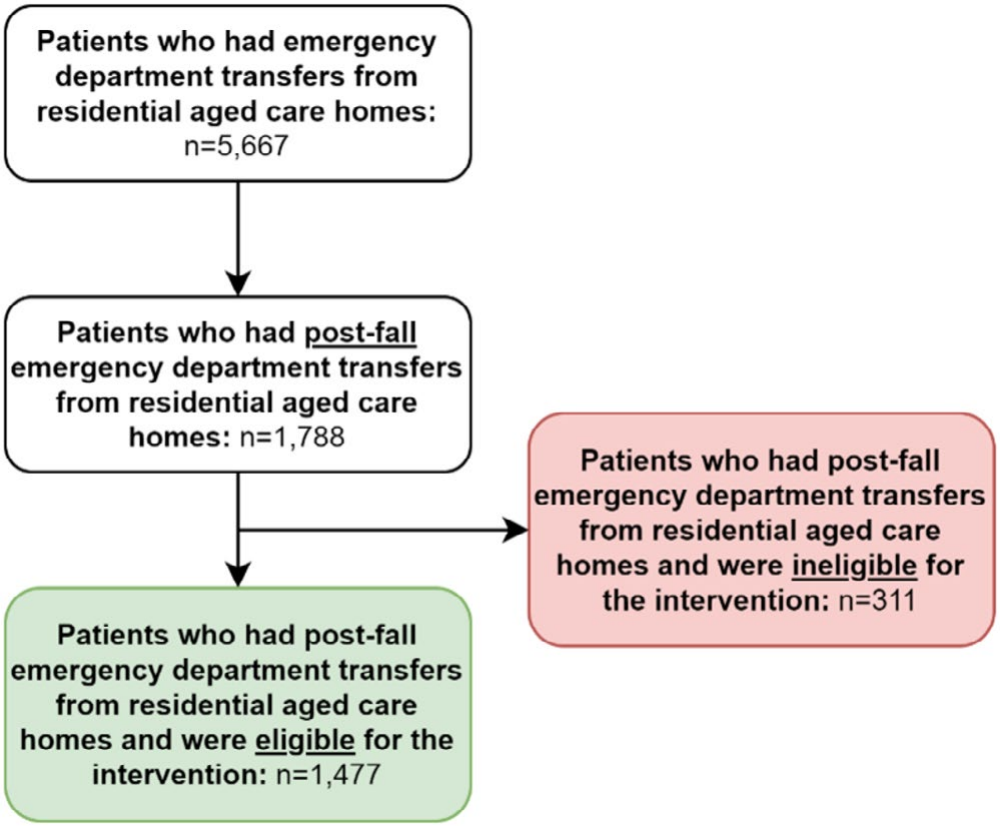
Study flow diagram.

A total of 5667 patients who had emergency department transfers from residential aged care homes were identified (Figure 1). Among patients who had post‐fall emergency department transfers from residential aged care homes (*n* = 1788), 1477 were deemed eligible for the intervention, and 311 were deemed ineligible for the intervention. Clinical characteristics of emergency department transfers from residential aged care homes are shown in Table 1. Among post‐fall transfers eligible for the intervention, most were female, few identified as Aboriginal or Torres Strait Islander, few required an interpreter, most arrived by ambulance, age ranged from 54 to 105 years, and emergency department length of stay ranged from 0.1 to 65.5 h.

**TABLE 1:** Clinical characteristics of emergency department transfers from residential aged care homes.

	All transfers (*n* = 5667)	Post‐fall transfers (*n* = 1788)	Post‐fall transfers among patients eligible for the intervention (*n* = 1477)	Post‐fall transfers among patients ineligible for the intervention (*n* = 311)
Age, years	85.4 (8.6)	87.2 (7.5)	87.0 (7.6)	87.8 (7.1)
Female, *n* (%)	3391 (59.8)	1176 (65.8)	943 (63.9)	233 (74.9)
Aboriginal or Torres Strait Islander, *n* (%)	22 (0.4)	2 (0.1)	2 (0.1)	0 (0.0)
Required an interpreter, *n* (%)	335 (5.9)	105 (5.9)	85 (5.8)	20 (6.4)
Arrived by ambulance, *n* (%)	5536 (97.7)	1763 (98.6)	1455 (98.5)	308 (99.0)
Emergency department length of stay, h	10.5 (6.8)	9.4 (5.9)	9.0 (5.8)	11.0 (6.1)

*Note:* Data are mean (SD) or count (percentage within group).

### 3.2 Outcome Data and Main Results

**FIGURE 2:**
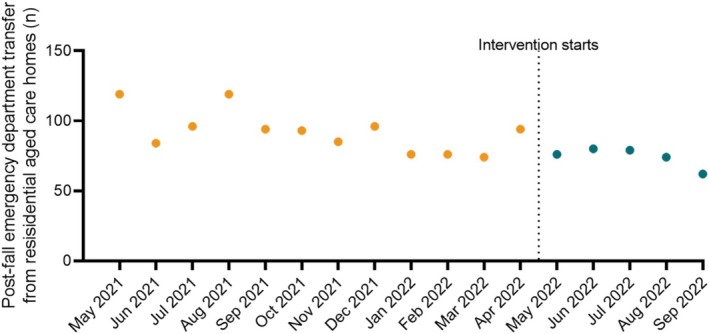
Monthly post‐fall emergency department transfer from residential aged care homes among patients eligible for the intervention.

Monthly post‐fall emergency department transfers from residential aged care homes among patients eligible for the intervention are shown in Table 2 and Figure 2. Mean (SD) monthly post‐fall emergency department transfers from residential aged care homes among patients eligible for the intervention were 92.2 (14.9) before and 74.2 (7.2) after the intervention (mean difference [95% CI]: 18.0 [2.9, 33.1]; *p* = 0.023).

**TABLE 2:** Monthly post‐fall emergency department transfers from residential aged care homes.

	Patients eligible for the intervention	Patients ineligible for the intervention
Pre‐intervention		
May 2021	119	15
June 2021	84	14
July 2021	96	18
August 2021	119	27
September 2021	94	20
October 2021	93	24
November 2021	85	18
December 2021	96	16
January 2022	76	17
February 2022	76	13
March 2022	74	21
April 2022	94	23
Post‐intervention		
May 2022	76	13
June 2022	80	14
July 2022	79	20
August 2022	74	15
September 2022	62	23

*Note:* Data are count.

### 3.3 Other Analyses

Monthly post‐fall emergency department transfers from residential aged care homes among patients ineligible for the intervention did not differ significantly before (mean [SD]: 18.8 [4.3]) and after (17.0 [4.3]) the intervention (mean difference [95% CI]: 1.8 [−3.0, 6.7], *p* = 0.434).

When compared to a season‐matched before period, monthly post‐fall emergency department transfers from residential aged care homes among patients eligible for the intervention were lower after the intervention (mean difference [95% CI]: 28.2 [10.3, 46.1], *p* = 0.007). When compared to a season‐matched before period, monthly emergency department transfers from residential aged care homes among patients ineligible for the intervention did not differ significantly after the intervention (mean difference [95% CI]: 1.8 [−5.1, 8.7], *p* = 0.566).

## 4 Discussion

The current study showed that monthly post‐fall emergency department transfers from residential aged care homes among patients eligible for an intervention that enabled the provision of on‐site assessment and management were lower after implementation. Sensitivity analyses supported the robustness of this estimate. Specifically, there was no difference in monthly post‐fall emergency department transfers from residential aged care homes among patients ineligible for the intervention, and seasonality did not appear to moderate effectiveness.

Using routinely collected administrative datasets, we detected a drop in preventable residential aged care home transfers of 90 (78% [95% CI: 70%, 85%]) during a period where there were 116 verified post‐fall emergency department transfers in this cohort prevented by our intervention [8]. When compared to a systematic review of 15 studies that used routinely collected administrative datasets to identify *preventable* emergency department visits among patients of all ages, our accuracy was more than four‐fold greater than average (mean [95% CI]: 18.3% [11.1%, 25.6%]) [16] and approximately 1.6‐fold greater than the most accurate study (49.2% [48.7%, 49.7%]) [17]. Moreover, our accuracy was more than five‐fold greater than average (14.0% [8.0%, 20.0%]) [16] and almost two‐fold greater than the most accurate study (39.3% [37.4%, 41.2%]) [17] among patients aged ≥ 65 years. Given the comparatively high accuracy of our method for identifying prevented post‐fall emergency department transfers from residential aged care homes, evaluating this approach in other studies appears warranted.

Despite the promising accuracy of our method, myriad contextual factors may in part explain the discordance between verified post‐fall emergency department transfers from residential aged care homes prevented by our intervention and system‐level utilisation. Although our season‐matched sensitivity analysis supported that temporal fluctuations were unlikely to explain the observed reductions in emergency department transfers, unmeasured seasonal variation (e.g., winter surges in respiratory illness) [18] or broader system pressures (e.g., ambulance demand) [19] may still have impacted our observations. Furthermore, effects borne from regression to the mean [20] or contamination from concurrent organisational initiatives [21] cannot be excluded. Collectively, these considerations highlight the need for cautious interpretation, as well as further research that facilitates more complex modelling such as multiple‐group interrupted time series analysis [22].

Our observations that the intervention may have reduced system‐level emergency department utilisation align with conclusions from a scoping review [23] that showed favourable results from three [24, 25, 26] structured protocols for determining appropriateness of post‐fall emergency department transfer among residents from residential aged care homes. However, given that one study [24] omitted post‐fall emergency department transfer outcomes and the other two studies [25, 26] did not collect pre‐intervention data, the possibility of comparing temporal observations from these studies to those in the current study is limited. The burgeoning literature available, as well as the promising findings from our study, provide support for further investigation of structured protocols that aim to reduce post‐fall emergency department transfer from residential aged care homes in other health services.

Reducing emergency department transfers has marked benefits from both the health service and patient perspectives. Emergency departments are systemically overcrowded, whereby services cannot meet patient demand [27], and the 9.0 million Australians who receive emergency care each year are at increasing risk of delays and poor quality care [11]. A systematic review of 83 studies concluded that residents from residential aged care homes who attended emergency departments had a greater length of stay and likelihood of admission to short stay units compared to community‐dwelling older adults [28]. Moreover, these patients are susceptible to hospital‐acquired illness (e.g., delirium) and infection (e.g., gastrointestinal or respiratory tract) [28]. Per the clinical characteristics of the 1477 post‐fall emergency department transfers from residential aged care homes among patients eligible for the intervention in Table 1, our intervention may have reduced system‐level emergency department utilisation by 810 h total or 5.4 h per day across the 5‐month intervention period. Given the consequences of emergency department transfer among residents from residential aged care homes, it is likely that observations from our study are clinically meaningful.

A strength of our study was expanding the scope of an existing service (Eastern Health Residential Inreach Service) [13], rather than the creation of a new service, which increases the feasibility of translating our findings into usual care. Examining whether our intervention could yield similar results when embedded within other established services, such as the virtual component of the New South Wales Aged Care Rapid Response Team [7], appears warranted. Another strength was conducting the current study within a real‐world setting that enabled examination of effectiveness, rather than efficacy [29], and thus markedly heightened ecological validity. However, our study had several limitations. First, there was no independent control group, such as another health service or cluster of residential aged care homes, which limited our capacity to control for potential confounding variables. Second, we only included residential aged care homes affiliated with one health service, which despite comprising 37 more sites than the collective 71 sites across the three prior studies [24, 25, 26] that examined similar post‐fall structured protocols, may limit generalisability. For example, the intervention may vary across residential aged care homes with different staffing structures, clinical skill mix, or access to other healthcare services [30]. Third, the administrative datasets used may be subject to coding errors. Fourth, we only included clinical characteristics available via routinely collected administrative datasets; thus, the extent of clinical heterogeneity among our sample remains unknown. Future studies could benefit from including fall mechanisms, associated injuries, functional baseline, and comorbidity profiles, noting that many of these fields would not typically be available in routinely collected administrative datasets. Finally, our intervention was only operational during daytime hours, and while inclusion was limited to patients who experienced a fall within the last 24 h, the heightened prevalence of falls among older adults during the night [31] may have moderated our estimates.

## 5 Conclusion

On‐site assessment and management following a fall at residential aged care facilities may be a viable strategy for reducing system‐level emergency department utilisation. Evaluation in other health services, as well as examination of sustainability and cost‐effectiveness, appears warranted.

## Author Contributions

**Patrick J. Owen:** data curation, formal analysis, software, visualisation, writing – original draft, methodology, writing – review and editing, approved final manuscript. **Andrea Bee:** investigation, methodology, writing – review and editing, approved final manuscript. **Donna Pattison:** investigation, methodology, writing – review and editing, approved final manuscript. **Joseph Miller:** investigation, methodology, writing – review and editing, approved final manuscript. **Liam Hackett:** investigation, methodology, writing – review and editing, approved final manuscript. **Paul Buntine:** conceptualisation, data curation, funding acquisition, investigation, methodology, project administration, resources, validation, visualisation, writing – original draft, writing – review and editing, approved final manuscript.

## Funding

The *Falls Outreach and Residential Mobile Assessment Team* (FORMAT) was implemented by Eastern Health Residential Inreach Service as part of the Victorian Department of Health Residential Inreach Surge Capacity Funding to the North East Metro Health Service Partnership. Evaluation was supported by the Society to Improve Diagnosis in Medicine (grant number: 604).

## Conflicts of Interest

The authors declare no conflicts of interest.

## Supporting information


**Supplementary A** STROBE statement.
**Supplement B**. Emergency medicine educational training on head injuries and wound management.

## Data Availability

The data that support the findings of this study are available on request from the corresponding author. The data are not publicly available due to privacy or ethical restrictions.
