# Supplementary material for: Reducing Post‐Fall Emergency Department Transfer From Residential Aged Care Homes: The Falls Outreach and Residential Mobile Assessment Team (FORMAT) Before‐and‐After Study
Source: Emerg Med Australas. 2026 May 18;38:e70276. doi: 10.1111/1742-6723.70276 (PMC13183477; doi:10.1111/1742-6723.70276)
Supplement: Supplementary file 1 — Supplementary A STROBE statement. Supplement B. Emergency medicine educational training on head injuries and wound management. [file EMM-38-0-s001.docx]

**SUPPLEMENT**

**Supplement A**. STROBE statement.

| **Section/Topic** | # | Reporting item | Reported |
| --- | --- | --- | --- |
| **Title and abstract** | 1 | (a) Indicate the study’s design with a commonly used term in the title or the abstract | TITLE |
|  |  | (b) Provide in the abstract an informative and balanced summary of what was done and what was found | ABSTRACT |
| Introduction | | |  |
| Background/rationale | 2 | Explain the scientific background and rationale for the investigation being reported | INTRODUCTION, Paragraph 1–2 |
| Objectives | 3 | State specific objectives, including any prespecified hypotheses | INTRODUCTION, Paragraph 3 |
| Methods | | |  |
| Study design | 4 | Present key elements of study design early in the paper | METHODS, Study design and setting |
| Setting | 5 | Describe the setting, locations, and relevant dates, including periods of recruitment, exposure, follow-up, and data collection |  |
| Participants | 6 | (a) Give the eligibility criteria, and the sources and methods of selection of participants. Describe methods of follow-up | METHODS, Participants |
|  |  | (b) For matched studies, give matching criteria and number of exposed and unexposed |  |
| Variables | 7 | Clearly define all outcomes, exposures, predictors, potential confounders, and effect modifiers. Give diagnostic criteria, if applicable | METHODS, Variables |
| Data sources/ measurement | 8* | For each variable of interest, give sources of data and details of methods of assessment (measurement). Describe comparability of assessment methods if there is more than one group | METHODS, Data sources and measurement |
| Bias | 9 | Describe any efforts to address potential sources of bias | METHODS, Bias |
| Study size | 10 | Explain how the study size was arrived at | METHODS, Study size |
| Quantitative variables | 11 | Explain how quantitative variables were handled in the analyses. If applicable, describe which groupings were chosen and why | METHODS, Quantitative variables |
| Statistical methods | 12 | (a) Describe all statistical methods, including those used to control for confounding | METHODS, Statistical methods |
|  |  | (b) Describe any methods used to examine subgroups and interactions |  |
|  |  | (c) Explain how missing data were addressed |  |
|  |  | (d) If applicable, explain how loss to follow-up was addressed |  |
|  |  | (e) Describe any sensitivity analyses |  |
| Results | | |  |
| Participants | 13* | (a) Report numbers of individuals at each stage of study—eg numbers potentially eligible, examined for eligibility, confirmed eligible, included in the study, completing follow-up, and analysed | RESULTS, Participants and descriptive data |
|  |  | (b) Give reasons for non-participation at each stage |  |
|  |  | (c) Consider use of a flow diagram |  |
| Descriptive data | 14* | (a) Give characteristics of study participants (eg demographic, clinical, social) and information on exposures and potential confounders |  |
|  |  | (b) Indicate number of participants with missing data for each variable of interest |  |
|  |  | (c) Summarise follow-up time (eg, average and total amount) |  |
| Outcome data | 15* | Report numbers of outcome events or summary measures over time | RESULTS, Outcome data and main results |
| Main results | 16 | (a) Give unadjusted estimates and, if applicable, confounder-adjusted estimates and their precision (eg, 95% confidence interval). Make clear which confounders were adjusted for and why they were included |  |
|  |  | (b) Report category boundaries when continuous variables were categorized |  |
|  |  | (c) If relevant, consider translating estimates of relative risk into absolute risk for a meaningful time period |  |
| Other analyses | 17 | Report other analyses done—eg analyses of subgroups and interactions, and sensitivity analyses | RESULTS, Other analyses |
| Discussion | | |  |
| Key results | 18 | Summarise key results with reference to study objectives | DISCUSSION, Paragraph 1 |
| Limitations | 19 | Discuss limitations of the study, taking into account sources of potential bias or imprecision. Discuss both direction and magnitude of any potential bias | DISCUSSION, Paragraph 5 |
| Interpretation | 20 | Give a cautious overall interpretation of results considering objectives, limitations, multiplicity of analyses, results from similar studies, and other relevant evidence | DISCUSSION, Paragraph 2–4 |
| Generalisability | 21 | Discuss the generalisability (external validity) of the study results | DISCUSSION, Paragraph 2–5 |
| Other information | | |  |
| Funding | 22 | Give the source of funding and the role of the funders for the present study and, if applicable, for the original study on which the present article is based | DECLARATIONS, Funding |

*Give information separately for exposed and unexposed groups.

**Supplement B.** Emergency medicine educational training on head injuries and wound management.

To ensure consistent clinical standards and enhance multidisciplinary care delivery, staff participating in the FORMAT initiative received a structured learning package comprising the following modules:

**Learning package components**

- **Introduction to local process and definitions:** Overview of site-specific protocols and terminology relevant to the Inreach model.
- **Inclusion criteria:** Criteria guiding patient eligibility for referral and intervention.
- **Consent process:** Ethical and procedural guidelines for obtaining informed consent.
- **Falls assessment during referral:** Evaluation of fall-related risk factors and incident context at point of referral.
- **Trauma assessment and injury management:** Systematic approach to identifying and managing acute injuries.
- **Identifying triggers for a fall:** Clinical reasoning to uncover physiological, environmental, or medication-related causes.
- **Medication review**  Assessment of polypharmacy, adverse effects, and deprescribing opportunities.
- **Wound assessment and use of local anaesthetic:** Techniques for evaluating wound severity and administering anaesthesia.
- **Wound closure techniques:** Instruction in simple wound closure including suturing methods, stapling , gluing and closure strategies appropriate to wound type.
- **Post-closure wound care:** Guidelines for dressing, monitoring, and follow-up care.
- **Data reporting:** Documentation standards and reporting mechanisms for clinical outcomes.

**Education Sessions**

Staff attend recurring education sessions led by an emergency department consultant (FACEM) or emergency nurse practitioner. These sessions were held every 3–5 months and aligned with the rotation of new registrars into the Inreach program. The sessions reinforced clinical competencies and promoted interdisciplinary learning.

**Reference Materials**

MiniMedLessons. *SUTURE Tutorial: Simple Interrupted Suture - Step-by-step instruction in HD!* YouTube; 2016. Accessed 12 November 2025. https://www.youtube.com/watch?v=pGEJEUZFIEk

VATA. *Suture Techniques Course Video*. YouTube; 2017. Accessed 12 November 2025. https://www.youtube.com/watch?v=Akyr4zlBS9E

Surgical Teaching. *How to Close a Wound with Surgical Staples*. YouTube; 2021. Accessed 12 November 2025. https://www.youtube.com/watch?v=ej0-2SyeRL4

SnareMan. *Quick Medical Tip: Skin Glue (Dermabond)*. YouTube; 2015. Accessed 12 November 2025. https://www.youtube.com/watch?v=vDLrxILRwfA
